# Effect of renal embolization in patients with synchronous metastatic renal cell carcinoma: a retrospective comparison of cytoreductive nephrectomy and systemic medical therapy

**DOI:** 10.18632/oncotarget.17865

**Published:** 2017-05-15

**Authors:** Sung Han Kim, Jung Kwon Kim, Boram Park, Jungnam Joo, Jae Young Joung, Ho Kyung Seo, Kang Hyun Lee, Jinsoo Chung

**Affiliations:** ^1^ Department of Urology, Center for Prostate Cancer, Research Institute and Hospital of National Cancer Center, Goyang, Korea; ^2^ Biometrics Research Branch, Division of Cancer Epidemiology and Prevention, Research Institute and Hospital of National Cancer Center, Goyang, Korea

**Keywords:** renal cell carcinoma, metastasis, synchronous, embolization, nephrectomy

## Abstract

**Objective:**

To compare survival outcomes for renal embolization (RE) to cytoreductive nephrectomy (CN) and no primary renal treatment (NT) among patients with synchronous metastatic renal cell carcinoma (mRCC) treated using either targeted therapy (TT) or immunotherapy (IT).

**Results:**

The median follow-up duration was 81.3 months, with a duration of first-line treatment of 3.5 months. Among the 211 patients, the median PFS and OS were 4.4 and 10.6 months. Specifically for patients receiving TT (124 patients), the PFS and OS were 5.5 and 12.0 months. An intervention effect was identified only for OS, with a median OS of 20.1, 8.8 and 9.3 months for CN, RE and NT, respectively. After stratification by risk classification, CN provided a significant benefit on OS, compared to RE and NT, for patients with an intermediate risk (MSKCC). For those with a poor risk (Heng criteria), NT provided better survival than PFS (*p*=0.003), and a comparable survival to RE (*p* > 0.05).

**Materials and Methods:**

Retrospective analysis of 211 patients, 87 treated with IT and 124 with TT, retrieved from our RCC database. Patients' risk factors for survival was evaluated using the Heng and MSKCC criteria, with only patients with an intermediate or poor survival risk included in the analysis. Between-group comparisons were evaluated with respect to progression-free survival (PFS) and overall survival (OS).

**Conclusions:**

The differential effect of CN and RE on OS appears to be modulated by risk classification. In patients with a poor risk, RE should be implemented after careful consideration of comorbidities and life expectancy.

## INTRODUCTION

The 5-year survival rate among patients with a newly diagnosed metastatic renal cell carcinoma (mRCC) or synchronous mRCC (smRCC) has traditionally been low at 0–20% [1, 2]. Recently, however, the disease-free survival and progression-free survival (PFS) of these patients has been remarkably prolonged by using targeted therapy (TT), which is based on patient-specific genome analysis, rather than conventional immunotherapy (IT) [2]. According to recent international guidelines for the treatment of mRCC, TT is regarded as the treatment of choice for first-line therapy, being supplemented, as needed, with other modalities including surgery and radiation [3]. With regard to surgical intervention, cytoreductive nephrectomy (CN) is regarded as the standard of care to improve the prognosis and quality of life of patients with mRCC, and in particular for those having low and intermediate risk for survival, large primary tumors, limited metastatic burden, and overall good functional status [4, 5]. However, in the absence of randomized clinical trials, controversy persists regarding the prognostic benefits of CN in the era of TT. Renal arterial embolization (RE) provides an alternative treatment option for high risk patients who have symptomatic unresectable mRCC or who are unsuitable for surgery due to multiple comorbidities and poor functional status [6, 7]. However, the efficacy of RE compared to that of CN in improving survival has yet to be evaluated. Therefore, the aim of our study was to evaluate the clinical effects of RE, CN and no primary renal treatment (NT) in patients with smRCC treated with systemic IT or TT as a first-line therapy. Our focus was on patients classified as being at intermediate or poor risk of survival, based on the International Metastatic Renal Cell Carcinoma Database Consortium (IMDC) classification, also known as the Heng criteria, and the Memorial Sloan Kettering Cancer Center (MSKCC) risk model. The primary outcomes were PFS and overall survival (OS).

## RESULTS

Relevant demographic data for our study group are summarized in Table 1. The median follow-up duration among the 211 patients in our study group was 81.3 (range, 4.8–147.6) months, with a median treatment duration of 3.5 (range, 1.0–70.4) months, a PFS of 4.4 (range, 1.0–70.4) months, and an OS of 10.6 (range, 1.0–139.6) months (Table 1). The MSKCC/Heng risk classification into intermediate and poor risk groups included 190/165 (90.0%/78.2%) and 21/46 (10.0%/21.8%) patients, respectively. For the 124 patients treated with TT, the median follow-up duration was 60.1 (range, 4.8–63.7) months, with a median treatment duration of 4.8 (range, 1.0–70.4) months. The PFS and OS were 5.5 (range, 1.0–70.4) and 12.0 (range, 1.0–97.1) months, respectively, with a MSKCC/Heng risk classification of 117/99 (94.4/79.8%) and 7/25(5.7/20.2%) into the intermediate and poor risk groups, respectively.

**Table 1:** Baseline characteristics

		*N* (%) or median (min–max)		
		Overall patients	Targeted therapy	Immunotherapy
Total		211	124	87
Age (years)		58 (13–80)	58 (34–80)	58 (13–76)
Sex	Male	167 (79.2)	99 (79.8)	68 (78.2)
	Female	44 (20.9)	25 (20.2)	19 (21.8)
Group	Embolization	29 (13.7)	13 (10.5)	16 (18.4)
	Nephrectomy	54 (25.6)	27 (21.8)	27 (31)
	No treatment	128 (60.7)	84 (67.7)	44 (50.6)
ECOG-PS*	0–2	185 (88.1)	105 (85.4)	80 (92)
	≥3	25 (11.9)	18 (14.6)	7 (8.1)
Karnofsky PS^	80–100	185 (94.4)	105 (92.1)	80 (97.6)
	≤70	11 (5.6)	9 (7.9)	2 (2.4)
Heng risk group	Intermediate	165 (78.2)	99 (79.8)	66 (75.9)
	Poor	46 (21.8)	25 (20.2)	21 (24.1)
MSKCC risk group	Intermediate	190 (90.0)	117 (94.4)	73 (83.9)
	Poor	21 (10.0)	7 (5.7)	14 (16.1)
Clinical Stage (T,N)	T1, T2, T3, T4, Tx	43/34/37/14/27	28/21/24/8/14	15/13/13/6/13
	N0, N1, Nx	67/39/49	41/29/25	26/10/24
Fuhrman nuclear grade	1	8 (3.9)	5 (4.2)	3 (3.5)
	2	33 (15.9)	21 (17.5)	12 (13.8)
	3	63 (30.4)	32 (26.7)	31 (35.6)
	4	37 (17.9)	26 (21.7)	11 (12.6)
	Unknown	66 (31.9)	36 (30.0)	30 (34.5)
Histology	Clear cell	159 (77.6)	98 (83.1)	61 (70.1)
	Non-clear cell	9 (4.4)	5 (4.2)	4 (4.6)
	Unknown	37 (18.1)	15 (12.7)	22 (25.3)
First-line immunotherapy		87 (41.2)		
Treatment duration (month)		3.5 (1.0–70.4)	4.8 (1.0–70.42)	2.6 (1.0–24.0)
Follow-up duration (month)		81.3 (4.8–147.6)	60.1 (4.8–63.7)	141.5 (56.3–147.6)
Progression free survival (month)		4.4 (1.0–70.4)	5.5 (1.0–70.4)	3.2 (1.0–24.0)
Overall survival (month)		10.6 (1.0–139.6)	12.0 (1.0–97.1)	9.6 (1.0–139.6)
Cancer specific survival (month)		10.7 (1.0–139.6)	12.0 (1.0–97.1)	9.8 (1.0–139.6)

ECOG-PS*: Eastern Cooperative Oncology Group Performance Status.

Karnofsky-PS^: Karnofsky Performance Status.

**Figure 1:**
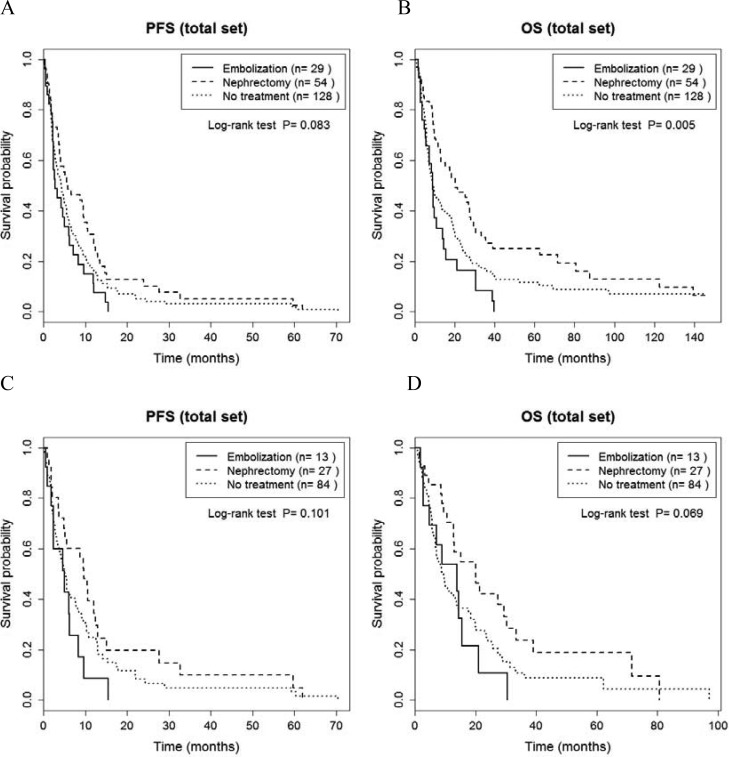
Comparison of progression-free survival (**A**, **C**) and overall survival (**B**, **D**) between cytoreductive nephrectomy, renal embolization and no invasive renal treatment groups, for the entire sample of 211 patients (A, B) and for the 124 patients treated with targeted therapy (C, D).

Regarding metastatic tumor burden, risk criteria and other prognostic factors summarized in Table 2, a between-group difference in risk criteria was identified (*P* < 0.05), with the CN group having only patients with an intermediate risk classification. Regarding survival among all 211 patients (Figure 1A, Supplementary Table 1), a significant between-group difference was identified for OS (*P* = 0.005; Figure 1B, Supplementary Table 1), but not PFS (*P* = 0.083, Figure 1A, Supplementary Table 1). The median PFS and OS, respectively, for the three groups were as follows: CN group, 5.9 and 20.1 months; RE, 2.7 and 8.8 months; and NT, 4.2 and 9.3 months. For the subgroup of 112 TT patients, no between-group differences were identified (*P* > 0.05, Figure 1C and 1D; Supplementary Table 2), with the group median OS and PFS, respectively, as follows: CN, 20.1 and 9.7, months; RE, 13.8 and 5.1 months; NT, 9.4 and 5.2 months.

**Table 2:** Comparison between the three intervention groups

		Total	Embolization	Nephrectomy	No treatment	*p*-value
Overall systemic treated patients						
N		211	29	54	128	
Survival		30	2 (6.9)	9 (16.7)	19 (14.8)	0.454+
First-line systemic failure		183	27 (93.1)	44 (81.5)	112 (87.5)	0.304+
Heng risk group	Intermediate	165	15 (51.7)	54 (100.0)	96 (75.0)	<.001+
	Poor	46	14 (48.3)	0 (0.0)	32 (25.0)	
MSKCC risk group	Intermediate	190	23 (79.3)	54 (100.0)	113 (88.3)	0.006+
	Poor	21	6 (20.7)	0 (0.0)	15 (11.7)	
ECOG-PS^#^	0–2	185	27 (93.1)	43 (81.1)	115 (89.8)	0.172+
	≥ 3	25	2 (6.9)	10 (18.9)	13 (10.2)	
Karnofsky PS^^^	80–100	185	27 (96.4)	43 (93.5)	115 (94.3)	1.000*
	≤ 70	11	1 (3.6)	3 (6.5)	7 (5.7)	
Lung metastasis		154	24 (82.8)	37 (69.8)	93 (72.7)	0.431+
Liver metastasis		38	7 (24.1)	11 (20.8)	20 (15.8)	0.488+
Bone metastasis		65	5 (17.2)	20 (37.7)	40 (31.5)	0.157+
Brain metastasis		20	3 (10.7)	4 (7.6)	13 (10.6)	0.814+
Only targeted therapy treated patients						
N		124	13	27	84	
Survival		20	2 (15.4)	5 (18.5)	13 (15.5)	0.928*
First-line systemic failure		105	12 (92.3)	23 (85.2)	70 (83.3)	0.925*
Heng risk group	Intermediate	99	9 (69.2)	27 (100.0)	63 (75.0)	0.011+
	Poor	25	4 (30.8)	0 (0.0)	21 (25.0)	
MSKCC risk group	Intermediate	117	12 (92.3)	27 (100.0)	78 (92.9)	0.374*
	Poor	7	1 (7.7)	0 (0.0)	6 (7.1)	
ECOG-PS^#^	0–2	105	12 (92.3)	17 (65.4)	76 (90.5)	0.009*
	3–6	18	1 (7.7)	9 (34.6)	8 (9.5)	
Karnofsky PS^^^	80–100	105	12 (100.0)	17 (85.0)	76 (92.7)	0.377*
	≤ 70	9	0 (0.0)	3 (15.0)	6 (7.3)	
Lung metastasis		91	11 (84.6)	20 (74.1)	60 (71.4)	0.603+
Liver metastasis		26	4 (30.8)	7 (25.9)	15 (18.1)	0.458+
Bone metastasis		43	3 (23.1)	10 (37.0)	30 (36.1)	0.635+
Brain metastasis		15	2 (16.7)	2 (7.4)	11 (13.6)	0.623*

+, Pearson's Chi-square test; *, Fisher's exact test;

ECOG-PS^#^: Eastern Cooperative Oncology Group Performance Status

Karnofsky-PS^: Karnofsky Performance Status.

**Figure 2:**
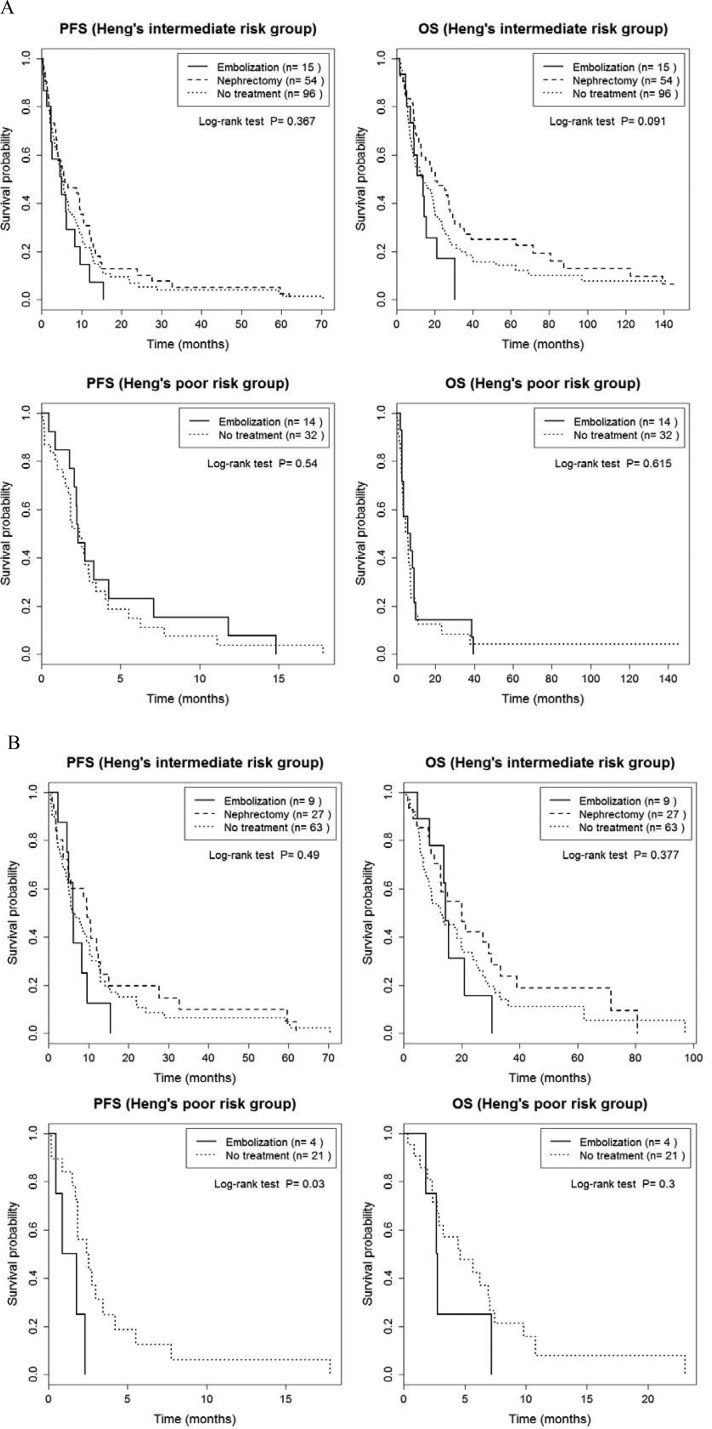
Comparison of progression-free survival and overall survival between cytoreductive nephrectomy, renal embolization and no invasive renal treatment groups, stratified by Heng's intermediate and poor risk classification, for the entire sample of 211 patients (**A**) and for the 124 patients treated with targeted therapy (**B**).

**Figure 3:**
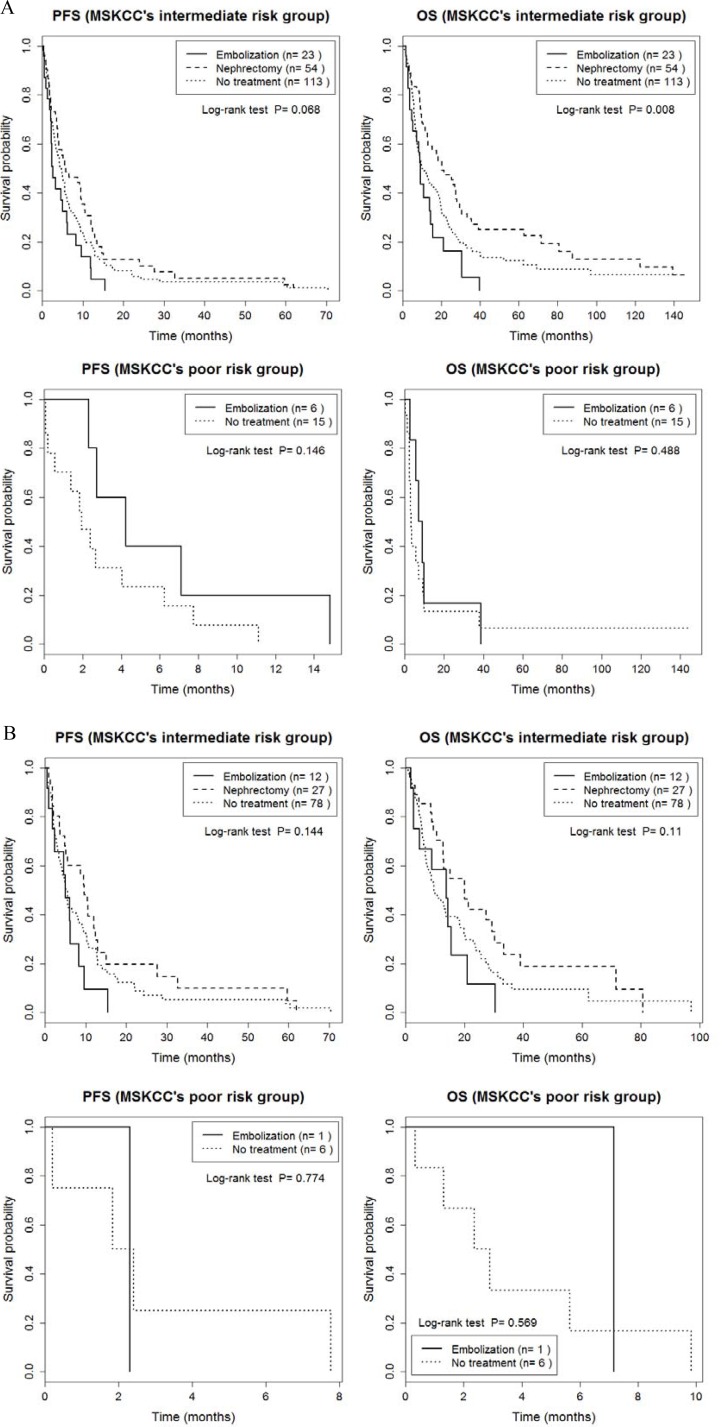
Comparison of progression-free survival and overall survival between cytoreductive nephrectomy, renal embolization and no invasive renal treatment groups, stratified by the MSKCC intermediate and poor risk classification, a for the entire sample of 211 patients (**A**) and for the 124 patients treated with targeted therapy (**B**).

Outcomes of our analysis of PFS and OS for patients stratified by Heng's and the MSKCC risk criteria are summarized in Supplementary Tables 1 and 2. PFS and OS were also comparable between groups when patients were stratified for risk using Heng's classification (*p* > 0.05, Figure 2A, Supplementary Table 1). When only patients who received TT were considered, there was a significant group effect on PFS for patients in Heng's poor risk group (*P* = 0.03; RE, 1.3 months; NT, 2.4 months, Figure 2B and Supplementary Table 2), with no effect of risk classification on OS (*p* > 0.05). When patients were stratified into intermediate and poor risks groups using the MSKCC classification, no significant between-group difference was identified for PFS, either for all 211 patients or for the subgroup of 112 patients who received TT (*P* > 0.05; Figure 3 and Supplementary Table 1). However, a significant between-group difference on OS was identified among all 211 patients in the intermediate risk group (*P* = 0.008; CN, 20.1 months; RE, 9.1 months; NT, 10.1 months; Figure 3A and Supplementary Table 1). Overall, patients in the CN group had the best prognostic outcome for PFS and OS, regardless of risk classification.

## DISCUSSION

Current international guidelines recommend TT as the treatment of choice for smRCC, in combination with interventional procedures as needed [3, 8]. RE can be used to treat patients with large unresectable smRCC, intravenocaval thrombosis or extensive lymphadenopathy, with outcomes expected to be comparable to those obtained with CN [7, 9]. The RE procedure can be performed either preoperatively to facilitate surgery or as palliative treatment to control symptoms, such as pain and hematuria [6, 10]. RE is also indicated for patients with a short life expectancy, due to major comorbidities or poor general condition, and for those unsuitable for general anesthesia [11, 12]. RE has the advantage over CN of being associated with a lower rate of major morbidity, as well as decreasing the delay between the procedure and initiation of TT [7, 9, 11, 13]. The lower morbidity rate with RE is clinically significant, with previous studies having reported that systemic therapy cannot be provided to 5.4–21.4% of patients after nephrectomy due to procedure-related morbidity [14, 15]. In comparison, symptomatic morbidity, due to post-embolic syndrome, has been reported in < 3% of RE cases, with symptoms, when present, being self-limiting [13, 16]. To date, however, any benefits of RE on the survival of patients with smRCC treated with TT have not been evaluated. In order to address this issue, we compared PFS and OS for RE, CN and NT, stratified by MSKCC and Heng risk criteria, with the aim of assisting clinicians in selecting the most feasible and best procedures to combine to TT.

When combined with IT, there is no survival benefit of RE, with or without subsequent CN [17], and survival could even be lower, as reported by Demirci et al. : median survival for RE of 1 (range, 1–74) months compared to 11 (range, 1–80) months for CN [18]. Although RE improved survival from 229 days to 7 months, compared to NT, survival was still lower than the 17.8 months for palliative nephrectomy [19, 20]. In our dataset (Supplementary Table 1), the longest survival among patients with an intermediate risk classification was achieved with CN, followed by NT and RE, regardless of IT or TT treatment. Notably, using a log-rank test (Supplementary Table 1), we did identify a significant difference in survival between RE and CN (9.1 *versus* 20.1 months, *p* = 0.002), and NT and CN (10.1 *versus* 20.1 months, *p* = 0.028), but with no difference between RE and NT (9.1 *versus* 10.1 months, *p* = 0.119). Our findings were comparable or superior to the OS previously reported for RE performed in patients treated with IT [19, 20]. We also identified an improved PFS among all patients receiving TT rather than IT, regardless of the procedural intervention (*p* < 0.001, Supplementary Figure 1). Improved survival reflects improvement in all prognostic indices of survival with TT, in combination with technical improvements, including imaging modalities, and improvement in embolic therapy used during RE [16, 21].

A priori, we had hypothesized that survival with RE would be comparable or even better than with CN, regardless of patient risk classification. Our hypothesis was based on evidence of an association between RE and a sustained systemic immunological response that would slow down disease progression in patients receiving IT treatment [10, 22]. Specifically, RE augments natural killer cell activity, B and T lymphoproliferative cell responses and immunomodulator agents, with these immunological responses, which would inhibit micrometastasis, persisting for up to 1-year post-RE [23]. Moreover, there is evidence that TT would synergistically enhance these effects of RE. RE produces complete obstruction of the blood supply to the primary tumor, including peripheral collateral devascularization. Further inhibition of the neovascularization by TT could enhance the therapeutic effects of RE in tumor destruction [10, 17, 22]. Rassweiler et al. showed that capillary or peripheral vessel occlusion resulted in complete coagulation necrosis of the kidney in healthy rat and canine models, an effect which is similar to nephrectomy and superior to main renal arterial occlusion [24]. In studies on the treatment of hepatocellular carcinoma using sorafenib in combination with transarterial embolization, the main effects of sorafenib on hepatic arterial occlusion, in combination with its anti-angiogenic and anti-proliferative effects, improved survival outcomes [25]. Moreover, the therapeutic effects of RE could allow a reduction in the therapeutic dosage of TT, which would increase patients' tolerability to TT and decrease the rate of adverse events, and, therefore, be of benefit, particularly in patients with a poor general health status [25, 26]. However, complete renal infarction of primary renal tumors is seldom achieved by RE [27, 28], with viability of the tumor being maintained by the induction of the collateral retroperitoneal vessels feeding the tumor and small-sized vessels on the periphery of the tumor [27, 28]. Incomplete tumor necrosis by RE would lower the survival of patients compared to complete removal of the kidney by CN [16, 21].

An important finding of our study was the effect of risk classification on survival prognosis. The overall OS showed significant differences between groups (*p* = 0.005, Figure 1B), whereas subgroup analyses of OS, with stratification based on the Heng and MSKCC risk criteria, did not identify between-group differences in OS (*p* > 0.05, Figures 2A and 3A). This absence of a difference based on risk classification can be explained by the low statistical power of our analysis of between-group differences, due to an insufficient sample size (Supplementary Table 1). Specifically, for patients with an intermediate risk classification (MSKCC), CN increased OS compared to RE (20.1 *versus* 9.1 months, respectively, *p* = 0.002; Figure 3A and Supplementary Table 1), but with no difference in PFS between these two groups. In addition, in the subgroup of TT patients with a poor Heng risk classification, PFS was significantly inferior for RE than NT (1.3 *versus* 2.4 months, respectively, *p* = 0.030; Figure 2B and Supplementary Table 2). Therefore, prompt surgical removal to decrease the overall tumor burden improved OS, with early initiation of systemic TT after CN being important to control disease progression [4, 29]. Importantly, for patients in the poor risk group who were not suitable for CN, RE did not provide a survival benefit.

Our finding of an overall lower survival with RE than with CN does not contraindicate the judicious use of RE for the treatment of smRCC. It is important to consider the significant between-group differences in baseline characteristics among patients who underwent RE compared to those who underwent CN (Table 2). Specifically, the rate of failure of first-line treatment was higher in the RE group (93.1% in IT group and 92.3% in TT group) than in the CN group (87.5% in IT group and 83.3% in TT group). Although this difference was not significant (*P* > 0.05, Table 2), it may have resulted in poorer outcomes in the RE group. However, survival outcomes after RE were not significantly different compared to those after NT among patients with a poor risk classification, regardless of systemic treatment received (Supplementary Tables 1 and 2), albeit with a uniquely lower PFS for patients classified using Heng's criteria and receiving TT (*P* = 0.03; Supplementary Table 2 and Supplementary Figure 2A). The insignificant benefit of RE over NT for patients with a poor risk receiving TT should be cautiously interpreted due to the small number of patients in each risk classification group. Future large sample studies are warranted to confirm our findings by controlling for baseline differences among the risk groups, to further clarify the modulation of the immunological response with RE and to determine if immune checkpoint inhibitors and tyrosine kinase inhibitors (TKIs) could further enhance this response, as recently suggested by the positive survival outcomes reported in phase 2 trials for nivolumab [30].

The limitations of our study need to be acknowledged. These include a retrospective design, short follow-up observation, small number of cases and between-group variation in baseline characteristics, including overall tumor burden, such as metastatic lesions and metastasectomy. Our median PFS of 4.4 months and OS of 10.6 months for all patients were comparable to previously reported survival rates for IT and TT therapy [2, 3, 5, 8]. As such, we deem our study to be clinically meaningful in comparing PFS and OS for CN, RE and NT interventions among patients treated using either systemic IT or TT as a first-line therapy, and by stratifying our analysis for risk, classified using the Heng and MSKCC criteria. Using this rigorous methodological approach, we do report better OS with CN than RE and NT for patients with an intermediate risk based on the MSKCC criteria. For patients classified as having a poor risk, RE and NT provided comparable survival. However, these insignificant benefits of RE compared to NT do not mean that RE should not be recommended in smRCC, when the inherent limitations of our study are considered. Consequently, prompt CN to decrease the overall tumor burden improved OS, with early initiation of systemic TT after CN being important to control disease, especially in patients with an intermediate risk of survival.

For patients with a poor risk of survival, RE should be implemented after careful consideration of comorbidities and life expectancy. Future studies are warranted to confirm our findings in a larger clinical group, as well as to determine the effect of metastatic tumor burdens and of metastasectomy.

## MATERIALS AND METHODS

Our retrospective study was approved by the Institutional Review Board of the National Cancer Center (IRB No. NCC 2016–0259), which waived the requirement for consent as all patient data were anonymized and de-identified prior to analysis.

Our study group consisted of the 270 patients with smRCC treated using IT or TT as a first-line therapy at our institution, between January 2000 and December 2015. Relevant medical data were extracted from our institution's prospective RCC database. Patients with no follow-up, incomplete medical records or who underwent both RE and CN were excluded. Among the 270 patients, 211 met our inclusion and exclusion criteria and formed our study group: 87 (41.2%) patients had received IT and 124 (58.8%) TT. Indications for RE were as follows: poor general condition and multiple comorbidities, including cardio/cerebrovascular disease, chronic medical renal disease and severe chronic hepatic disease; contra-indication to anesthesia or the CN procedure due to expected higher postoperative morbidity [9]; patient's reluctance to surgery; and symptomatic patients with unresectable smRCC, including gross hematuria and tumor-related pain.

For analysis, patients were classified into three groups according to the treatment modality received: CN with systemic IT or TT (*N* = 54, 25.6%); RE with systemic IT or TT group (*N* = 29, 13.7%); and no invasive primary renal procedure (NT) with patients treated only with systemic IT or TT (*N* = 128, 60.7%). An additional subgroup for analysis included only patients treated using TT as a first-line therapy (*N* = 124), in combination with RE (*N* = 13, 10.5%), CN (*N* = 27, 21.8%) and NT (*N* = 84, 67.7%). The pathological staging using Fuhrman's nuclear grade and the TNM staging for malignant tumors, based on the 2009 International Union Against Cancer Classification 2009, were recorded. Classification of risk stratification was based on the MSKCC and Heng clinical prognostic models. The RECIST criteria 1.1 were used to evaluate the response to systemic therapy, with the choice of IT and TT at the discretion of the treating urologist (J.C.), based on a patient's pathological profile and coverage guidelines of our national health insurance system.

### Statistical analysis

Baseline characteristics were summarized as frequency (percentage) for categorical variables and median (range) for continuous variables. Between-group differences were evaluated using Pearson's chi-squared test or Fisher's exact test, as appropriate for the dataset. PFS was defined from the time of initiation of first-line systemic therapy to the identification of disease progression. Cancer-specific survival (CSS) and OS were defined from the time of initiation of first-line systemic therapy to renal cell carcinoma-related death or any death, respectively. Between-group differences in PFS and OS were evaluated using log-rank test, with survival curves estimated using the Kaplan-Meier method. Treatment failure was defined as disease progression or unacceptable toxicity.

Subgroup analyses were performed using the MSKCC and Heng risk groups. The reverse Kaplan-Meier method was used to calculate the median follow-up duration. With this method, the median follow-up duration was estimated from the Kaplan-Meier method, but events are reversed. In addition, the event of interest here becomes ‘being alive', with ‘death’ being the censored event.

All statistical analyzes were performed using SAS (version 9.4; SAS Institute Inc., Cary, NC, USA) and R (version 3.3.0) software, with a *P*-value < 0.05 considered to be significant.

## SUPPLEMENTARY MATERIALS FIGURE AND TABLES



## Abbreviations

mRCC: renal cell carcinoma
smRCC: synchronous mRCC
RE: renal embolization
CN: cytoreductive nephrectomy
NT: no primary renal treatment
TT: targeted therapy
IT: immunotherapy
PFS: progression-free survival
OS: overall survival
CSS: Cancer-specific survival
