# Supplementary material for: Effect of renal embolization in patients with synchronous metastatic renal cell carcinoma: a retrospective comparison of cytoreductive nephrectomy and systemic medical therapy
Source: Oncotarget. 2017 May 15;8(30):49615–24. doi: 10.18632/oncotarget.17865 (PMC5564792; doi:10.18632/oncotarget.17865)
Supplement: Supplementary file 1 [file oncotarget-08-49615-s001.pdf]

Effect of renal embolization in patients with synchronous metastatic renal cell carcinoma: a retrospective comparison of cytoreductive nephrectomy and systemic medical therapy

Supplementary Materials

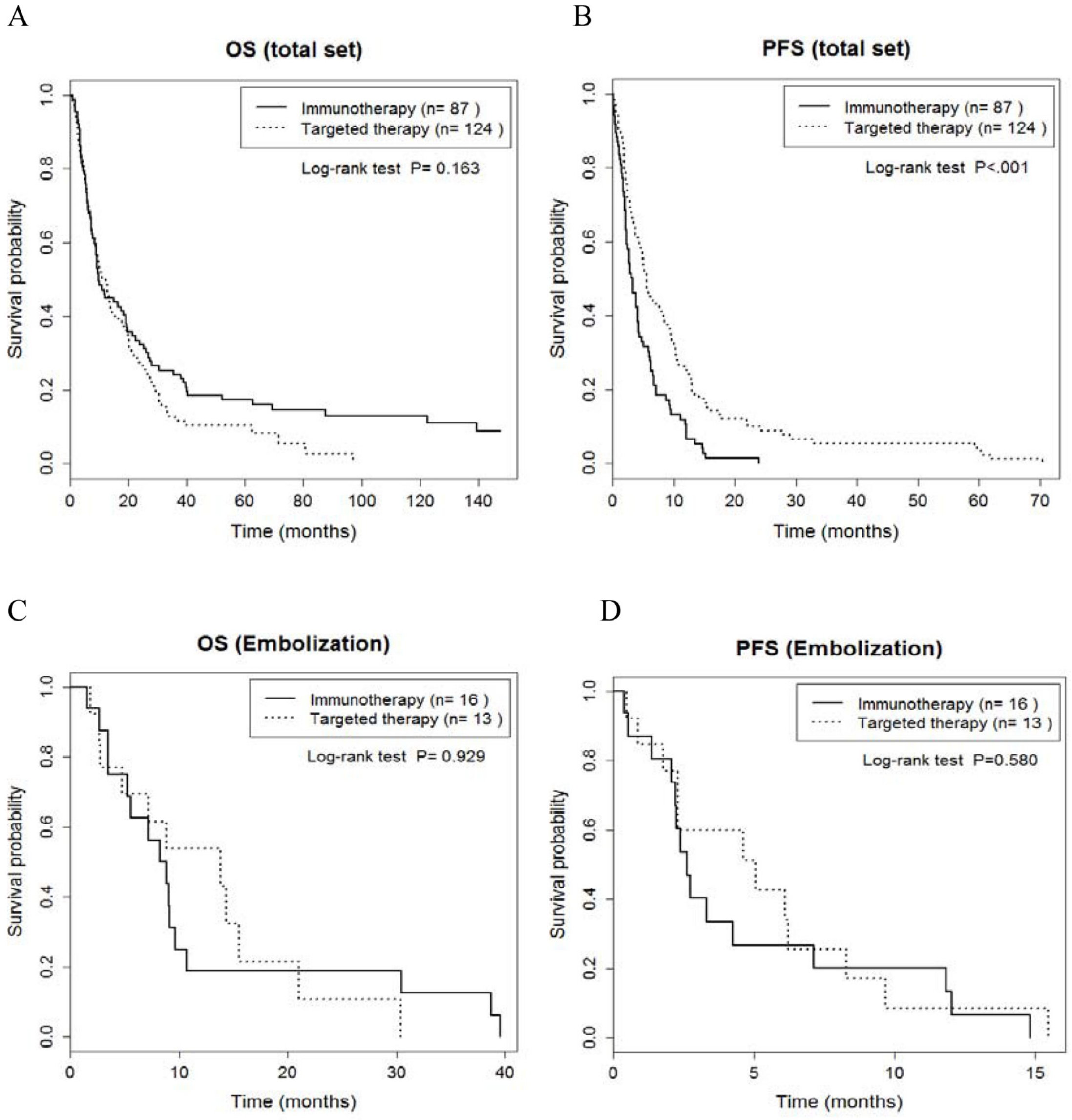

Supplementary Figure 1: Comparison of overall survival (**A**, **C**) and progression-free survival (**B**, **D**) between patients treated with immunotherapy ($n = 87$) and targeted therapy ($n = 124$) in the total patient group (A, B) and among patients in the embolization group only (C, D).

Supplementary Table1: Median survival time and 95% confidence interval for each group among overall 211 patients

Dataset		Group	Total	Event	median (months)	95% CI		Log-rank test p-value			
						Lower	Upper	three group	RE vs. CN	CN vs. NT	RE vs. NT
Total set	OS	Embolization	29	27	8.84	5.29	13.84	0.005	0.001	0.014	0.162
		Nephrectomy	54	45	20.12	12.72	27.91				
		No treatment	128	109	9.30	7.04	13.32				
	PFS	Embolization	29	27	2.73	2.24	6.08	0.083	0.029	0.109	0.258
		Nephrectomy	54	44	5.85	3.81	9.67				
		No treatment	128	112	4.21	2.99	5.52				
Heng risk group (intermediate)	OS	Embolization	15	13	13.84	5.29	21.01	0.091	0.035	0.107	0.296
		Nephrectomy	54	45	20.12	12.72	27.91				
		No treatment	96	80	13.58	8.65	19.33				
	PFS	Embolization	15	14	5.06	1.35	8.28	0.367	0.168	0.435	0.333
		Nephrectomy	54	44	5.85	3.81	9.67				
		No treatment	96	84	5.16	4.01	6.54				
Heng risk group (poor)	OS	Embolization	14	14	6.36	2.70	9.07	0.615	.	.	.
		No treatment	32	29	5.15	2.89	6.94				
	PFS	Embolization	14	13	2.30	1.78	4.24	0.540	.	.	.
		No treatment	32	28	2.40	1.51	3.06				
MSKCC risk group (intermediate)	OS	Embolization	23	21	9.07	4.77	14.37	0.008	0.002	0.028	0.119
		Nephrectomy	54	45	20.12	12.72	27.91				
		No treatment	113	95	10.06	7.53	17.59				
	PFS	Embolization	23	22	2.60	2.07	6.08	0.068	0.021	0.221	0.108
		Nephrectomy	54	44	5.85	3.81	9.67				
		No treatment	113	99	4.70	3.22	5.59				
MSKCC risk group (poor)	OS	Embolization	6	6	8.01	2.70	38.73	0.488	.	.	.
		No treatment	15	14	3.16	2.37	7.04				
	PFS	Embolization	6	5	4.24	2.30	14.83	0.146			
		No treatment	15	13	1.94	0.20	4.04				

Supplementary Table 2: Median survival time and 95% confidence interval for each group among 124 targeted therapy patients

Dataset		Group	Total	Event	median (months)	95% CI		Log-rank test p-value			
						Lower	Upper	three group	RE vs. CN	CN vs. NT	RE vs. NT
Total set	OS	Embolization	13	11	13.84	2.76	15.55	0.069	0.037	0.036	0.603
		Nephrectomy	27	22	20.12	10.59	30.35				
		No treatment	84	71	9.37	6.77	13.32				
	PFS	Embolization	13	12	5.06	1.78	8.28	0.101	0.026	0.151	0.234
		Nephrectomy	27	23	9.67	4.83	12.43				
		No treatment	84	70	5.16	3.39	7.69				
Heng risk group (intermediate)	OS	Embolization	9	7	14.37	4.77	21.01	0.377	0.302	0.187	0.898
		Nephrectomy	27	22	20.12	10.59	30.35				
		No treatment	63	52	13.32	8.65	19.82				
	PFS	Embolization	9	8	6.15	2.30	9.67	0.490	0.223	0.435	0.515
		Nephrectomy	27	23	9.67	4.83	12.43				
		No treatment	63	53	6.54	4.70	10.03				
Heng risk group (poor)	OS	Embolization	4	4	2.71	1.81	7.17	0.300	.	.	.
		No treatment	21	19	4.64	2.37	7.07				
	PFS	Embolization	4	4	1.32	0.46	2.30	0.030	.	.	.
		No treatment	21	17	2.40	1.71	3.45				
MSKCC risk group (intermediate)	OS	Embolization	12	10	13.84	2.66	21.01	0.110	0.060	0.065	0.530
		Nephrectomy	27	22	20.12	10.59	30.35				
		No treatment	78	65	9.63	7.07	17.59				
	PFS	Embolization	12	11	5.06	0.85	8.28	0.144	0.039	0.201	0.262
		Nephrectomy	27	23	9.67	4.83	12.43				
		No treatment	78	66	5.52	3.68	8.15				
MSKCC risk group (poor)	OS	Embolization	1	1	7.17	.	.	0.569	.	.	.
		No treatment	6	6	2.63	0.33	9.83				
	PFS	Embolization	1	1	2.30	.	.	0.774	.	.	.
		No treatment	6	4	2.12	0.20	7.76				
